# Whole-genome sequencing reveals Enterobacter hormaechei as a key bloodstream pathogen in six tertiary care hospitals in southwestern Nigeria

**DOI:** 10.1099/mgen.0.001508

**Published:** 2025-10-06

**Authors:** Faith I. Oni, Ayorinde Oluwatobiloba Afolayan, Anderson O. Oaikhena, Erkison Ewomazino Odih, Odion O. Ikhimiukor, Veronica O. Ogunleye, Aaron Oladipo Aboderin, Olatunde F. Olabisi, Adewale A. Amupitan, Abayomi Fadeyi, Rasaki A. Raheem, Bashirat A. Olanipekun, Charles J. Elikwu, Oluwadamilola A. Sadare, Philip O. Oshun, Oyinlola O. Oduyebo, Folashade Ojo, Abolaji T. Adeyemo, Ifeanyi E. Mba, Abiodun Egwuenu, Tochi J. Okwor, Anthony Underwood, Silvia Argimon, Chikwe Ihekweazu, David M. Aanensen, Iruka N. Okeke

**Affiliations:** 1 Global Health Research Unit for the Genomic Surveillance of Antimicrobial Resistance, Department of Pharmaceutical Microbiology, Faculty of Pharmacy, University of Ibadan, Ibadan, Oyo State, Nigeria; 2 Department of Medical Microbiology and Parasitology, University College Hospital, Ibadan, Oyo State, Nigeria; 3 Department of Medical Microbiology and Parasitology, Obafemi Awolowo University Teaching Hospitals Complex, Ile-Ife, Nigeria; 4 Department of Medical Microbiology and Parasitology, University of Ilorin, Ilorin, Kwara State, Nigeria; 5 Department of Medical Microbiology and Parasitology, University of Ilorin Teaching Hospital, Ilorin, Kwara state, Nigeria; 6 Department of Medical Microbiology, Babcock University Teaching Hospital, Ilishan-Remo, Ogun State, Nigeria; 7 Department of Medical Microbiology and Parasitology, Faculty of Basic Medical Sciences, College of Medicine, University of Lagos, Lagos, Nigeria; 8 Department of Medical Microbiology and Parasitology, UNIOSUN Teaching Hospital, Osogbo, Nigeria; 9 Nigeria Centre for Disease Control and Prevention, Jabi, Abuja, Nigeria; 10 Centre for Genomic Pathogen Surveillance, Big Data Institute, University of Oxford, Oxford, UK

**Keywords:** bacteraemia, bacterial identification, *Enterobacter*, *Enterobacter cloacae*, *Enterobacter hormaechei*, genomic surveillance

## Abstract

*Enterobacter* spp. are an important cause of healthcare-associated bloodstream infections that are uncommonly reported in Africa. This study utilized whole-genome sequencing to characterize *Enterobacter* spp. from hospitals in Nigeria’s antimicrobial resistance surveillance system. Bloodstream *Enterobacter* spp. isolates from six sentinel tertiary-care hospitals recovered between 2014 and 2020 were re-identified and antimicrobial susceptibility-tested using VITEK 2 system. Illumina technology provided whole-genome sequences for genome nomenclature, antimicrobial resistance gene prediction, SNP phylogeny and multi-locus sequence typing via publicly available bioinformatics pipelines. Initial biochemical delineation often misclassifies *Enterobacter*, necessitating whole-genome sequencing for accurate classification. Among 98 *Enterobacter* received, *Enterobacter hormaechei* subspecies *xiangfangensis* predominated (43), followed by other *E. hormaechei* subspecies (18) and *Enterobacter* spp. such as *Enterobacter cloacae* (26), *Enterobacter roggenkampii* (4), *Enterobacter bugandensis* (3), *Enterobacter kobei* (2), *Enterobacter asburiae* (1) and *Enterobacter cancerogenus* (1). Resistance to extended-spectrum cephalosporins, aminoglycosides, phenicols, macrolides and carbapenems in *E. hormaechei* was attributed to known resistance genes. *E. hormaechei* isolates belonged to clusters III, IV and VIII based on *hsp60* typing and clades A, B, C and D according to Sutton and Co’s nomenclature. This and other recent reports from Nigeria reveal the extensive diversity of *E. hormaechei*, as well as clusters representing potential outbreaks. *E. hormaechei*, often misidentified and rarely reported from Nigeria, is the most common *Enterobacter* spp. isolated from blood culture in this study. Uncovering underappreciated species as important bloodstream pathogens and retrospective detection of likely outbreaks emphasize the value of genomic surveillance in resource-limited settings.

**Impact Statement**

*Enterobacter* is a member of the ESKAPE group of clinically important pathogens that effectively escape antibiotic inhibitory action. Accurate identification of *Enterobacter* isolates is essential in healthcare settings, as misidentification can lead to the misuse of antimicrobial agents for empirical therapy, such as the selection of antimicrobials to which a genus is intrinsically resistant before susceptibility testing results are available. Also, misidentification can compromise microbiology support for infection prevention and control. We show that *Enterobacter hormaechei*, which has not been previously reported from clinical laboratories in Nigeria, is frequently misidentified using conventional manual and automated biochemical systems. Whole-genome sequence data demonstrate that *E. hormaechei* and *Enterobacter cloacae* are the most commonly isolated *Enterobacter* species from bloodstream infections in Nigeria. Enhanced identification methods for surveillance play a pivotal role in improving patient care, optimizing antibiotic stewardship and combating the evolving challenges posed by this pathogen. Overall, this study reveals the effectiveness of whole-genome sequencing in correctly identifying this important pathogen.

## Data Summary

All sequence reads were submitted to the European Nucleotide Archive under the project ID PRJEB29739 (https://www.ebi.ac.uk/ena/browser/view/PRJEB29739). Accessions are listed in Table S1.

## Introduction

The *Enterobacter* genus, comprising Gram-negative, non-spore-forming bacilli that belong to the *Enterobacteriaceae* family, is a natural commensal of the human and animal gut [1]. They are also commonly isolated from environmental sources such as water, sewage, soil and plants [2]. The genus is a member of the ‘ESKAPE’ group of pathogens (*Enterococcus*, *Staphylococcus*, *Klebsiella*, *Acinetobacter*, *Pseudomonas* and *Enterobacter* species), known to exhibit multidrug resistance often [3], and is an increasingly common cause of opportunistic nosocomial and community-acquired infections [3]. They are intrinsically resistant to ampicillin, amoxicillin, first-generation cephalosporins and cefoxitin due to the constitutive AmpC beta-lactamases (ACT beta-lactamases) [1]. *Enterobacter* can infect multiple sites, causing urinary tract infections, cerebral abscesses, wounds, pneumonia, meningitis, abdominal and surgical site infections [4], as well as bloodstream infections [4,5].

An important subgroup among the *Enterobacter* species is the *Enterobacter cloacae* complex (ECC), comprising six species: *Enterobacter cloacae*, *Enterobacter asburiae*, *Enterobacter hormaechei*, *Enterobacter kobei*, *Enterobacter ludwigii* and *Enterobacter nimipressuralis* [5]. These species often evade precise identification due to inaccurate biochemical classification using tube-based biochemicals, which is commonly used in resource-limited settings [6]. Automated biochemical systems, such as the VITEK 2 system and MALDI-TOF [5], also frequently misclassify *Enterobacter* species [7].

Whole-genome sequencing (WGS) emerges as a promising solution for precise species-level identification, as demonstrated in recent studies [7,8]. WGS enables a deeper understanding of ECC epidemiology, revealing cryptic species like *E. hormaechei* subsp. *xiangfangensis*, frequently misclassified as *E. cloacae* by traditional methods [9]. Notably, ECC is identified as a major Gram-negative bacterium responsible for neonatal sepsis in low- and middle-income countries, such as Nigeria [8]. However, many bloodstream-associated *Enterobacter* infections in Africa are reported with limited, if any, speciation or subspeciation data [10,12]. In Nigerian hospitals, necessary reliance on biochemical tests, particularly tube-based biochemicals, often results in misidentification and inadequate species-level resolution for this important antimicrobial resistance (AMR) priority pathogen. Nigeria’s National AMR surveillance system was launched in 2017, and the Nigerian arm of the Global Health Research Unit (GHRU) for genomic surveillance of AMR provides WGS-based reference laboratory services to sentinel laboratories across the country [13,14].

We used WGS to identify and characterise the prevalent *Enterobacter* species isolated from bloodstream infections in selected Nigerian hospitals between 2014 and 2020, uncovering the limitations of conventional biochemical methods.

## Methods

### Collection of presumptive *Enterobacter* species from the bloodstream

*Enterobacter* strains were isolated from blood cultures collected between 2014 and 2020 at six tertiary-care hospitals registered in the Nigerian AMR surveillance system. Metadata containing preliminary information for each isolate were received from the contributing hospitals. From a total of 2,383 isolates processed by the reference laboratory, 63 were presumptively identified by the sentinels as various species of *Enterobacter [E. cloacae* (*n*=29), *Enterobacter aerogenes* (*n*=27), *Enterobacter gergoviae* (*n*=5), *Enterobacter agglomerans* (*n*=1) and *E. hormaechei* (*n*=1)]. An additional 80 isolates not identified to species level were submitted as *Enterobacteriaceae*.

### Re-identification and antimicrobial susceptibility testing

Strains received through the antimicrobial resistance surveillance system were placed on MacConkey agar to assess colony purity and phenotype. Mixed cultures from heterogenous/polymicrobial infections were purified, and strains were re-identified using the VITEK 2 GN ID cards (21341). Their antimicrobial susceptibility profile was also determined using GN AST cards (N280 414531) that test for susceptibility to ampicillin, amikacin, gentamicin, cefuroxime, amoxicillin/clavulanic acid, cefepime, ceftriaxone, piperacillin/tazobactam, nitrofurantoin, cefuroxime_axetil, ciprofloxacin, nalidixic acid, meropenem, ertapenem, imipenem, tigecycline, trimethoprim-sulphamethoxazole and colistin. The results were interpreted according to the Clinical and Laboratory Standards Institute [15].

### DNA extraction, library preparation and whole-genome sequencing

DNA of isolates was extracted using the Wizard DNA Extraction Kit (Promega, Wisconsin, USA) (A1125) according to the manufacturer’s protocols. Extracted DNA was quantified using the Qubit dsDNA BR Assay Kit (Invitrogen, Waltham, MA, USA). Libraries were prepared using the NEBNext Ultra II FS DNA Library Kit for Illumina with 384 unique indexes (New England Biolabs, Ipswich, MA, USA). Double-stranded DNA libraries were then sequenced using the HiSeq X10 with 150-bp paired-end chemistry (Illumina, San Diego, CA, USA).

### Whole-genome sequence analysis

All sequence analyses were carried out using GHRU protocols (https://www.protocols.io/view/ghru-genomic-surveillance-of-antimicrobial-resista-bp2l6b11kgqe/v4). Genome assembly and quality control were carried out using the *de novo* assembly pipeline in the GHRU protocol. Assembly metrics were N50 score, >50,000; number of contigs that are ≥0 bp, <500; number of contigs that are ≥1,000 bp, <300; total length (≥ 1,000 bp), >4,096,846 or <6,099,522 and percentage_contamination <5.

Speciation and selection of reference for SNP phylogeny were done using the Bactinspector (check_species and closest_match) tool (https://gitlab.com/antunderwood/bactinspector). Pathogenwatch (https://pathogen.watch/ -v21.4.3 [16] was used to validate species identification. The closest reference genome selected for *E. hormaechei* was NZ_CP017183.1 (https://www.ncbi.nlm.nih.gov/nuccore/NZ_CP017183.1), while NZ_CP009756.1 (https://www.ncbi.nlm.nih.gov/nuccore/NZ_CP009756.1) was chosen for *E. cloacae*. Mapping to reference was done with the bwa mem tool (https://arxiv.org/abs/1303.3997). Variant calling and filtering were done with samtools/bcf tools (https://github.com/samtools/bcftools), and maximum likelihood phylogenetic trees were constructed. The pair-wise SNP distances for likely outbreak isolates were calculated using FastaDist (https://gitlab.com/antunderwood/fastadist).

Identification of multilocus sequence types (according to the Pasteur scheme) was done using the ARIBA software [17] and the PubMed database (https://www.protocols.io/view/ghru-genomic-surveillance-of-antimicrobial-resista-bpn6mmhe).

Antimicrobial resistance genes, virulence genes and plasmid replicons were predicted *in silico* using the aforementioned GHRU protocol. Predicted genes tagged as ‘yes’ or ‘yes_nonunique’ by the ARIBA software were accepted as present in the genomes. The criteria used for defining multidrug resistance in isolates, according to [18], are non-susceptibility to ≥1 agent in >3 antimicrobial categories [18]. AMRFinderPlus version 3.1.0 [19] and the Comprehensive Antimicrobial Resistance Database (CARD) [20] were used to determine *ampC* variants among *Enterobacter* spp.

Hoffman clustering of *Enterobacter* spp. was done using the hsp60ECC tool (https://github.com/karubiotools/hsp60ECCtool). Publicly available data from the Sands *et al.* [8] study were retrieved from the European Nucleotide Archive (ENA) under the project accession number SAMEA7472464. Fastq files of *Enterobacter* spp. isolated from Nigeria were downloaded from ENA (https://www.ebi.ac.uk/ena/browser/view/SAMEA7472464?show=reads) and assembled using the aforementioned GHRU *de novo* assembly protocol. Hoffman clustering and clades were determined for the species using the HSP60ECC tool.

The average nucleotide identity (ANI) of genomes was obtained using the FastANI tool (https://github.com/ParBLiSS/FastANI) [21]. The ‘many-to-many’ method in FastANI was used to compute ANI between multiple query genomes (genomes from this study) and multiple reference genomes (Sands *et al. Enterobacter* Nigerian genomes) [8].

Novel sequence types (STs) identified in this study were first confirmed as novel by querying their fasta sequences on the PubMLST public database for molecular typing and microbial genome diversity (https://pubmlst.org/bigsdb?db=pubmlst_ecloacae_seqdef). Their profiles were then submitted, and STs were assigned as follows: G20500026: assigned, ST-1995; G20500682: assigned, ST-1996; G18503215: assigned, ST-1997; G18503415: assigned, ST-1998; and G18503407: assigned, ST-1998. Simpson’s diversity index [22] was calculated using the R package vegan [23].

A pangenome analysis was performed using the Bacterial Pan-Genome Analysis Pipeline [24]. The expansion of the pangenome across functional categories was evaluated using Clusters of Orthologous Groups (COG) and Kyoto Encyclopedia of Genes and Genomes (KEGG) pathway analyses.

For data visualization, iTOL (https://itol.embl.de/tree/197211635839451656920611#) [25], itol.toolkit R package (https://github.com/TongZhou2017/itol.toolkit) [26] and Microsoft Excel version 16.62 (2022) were used.

For map drawing, R packages – naijR (https://docs.ropensci.org/naijR/articles/nigeria-maps.html), SF (https://cran.r-project.org/web/packages/sf/index.html), map (https://cran.r-project.org/web/packages/tmap/index.html) and feathers (https://cran.r-project.org/web/packages/feather/index.html) – were used.

## Results

### *Enterobacter* species identified by WGS

**Fig. 1.**
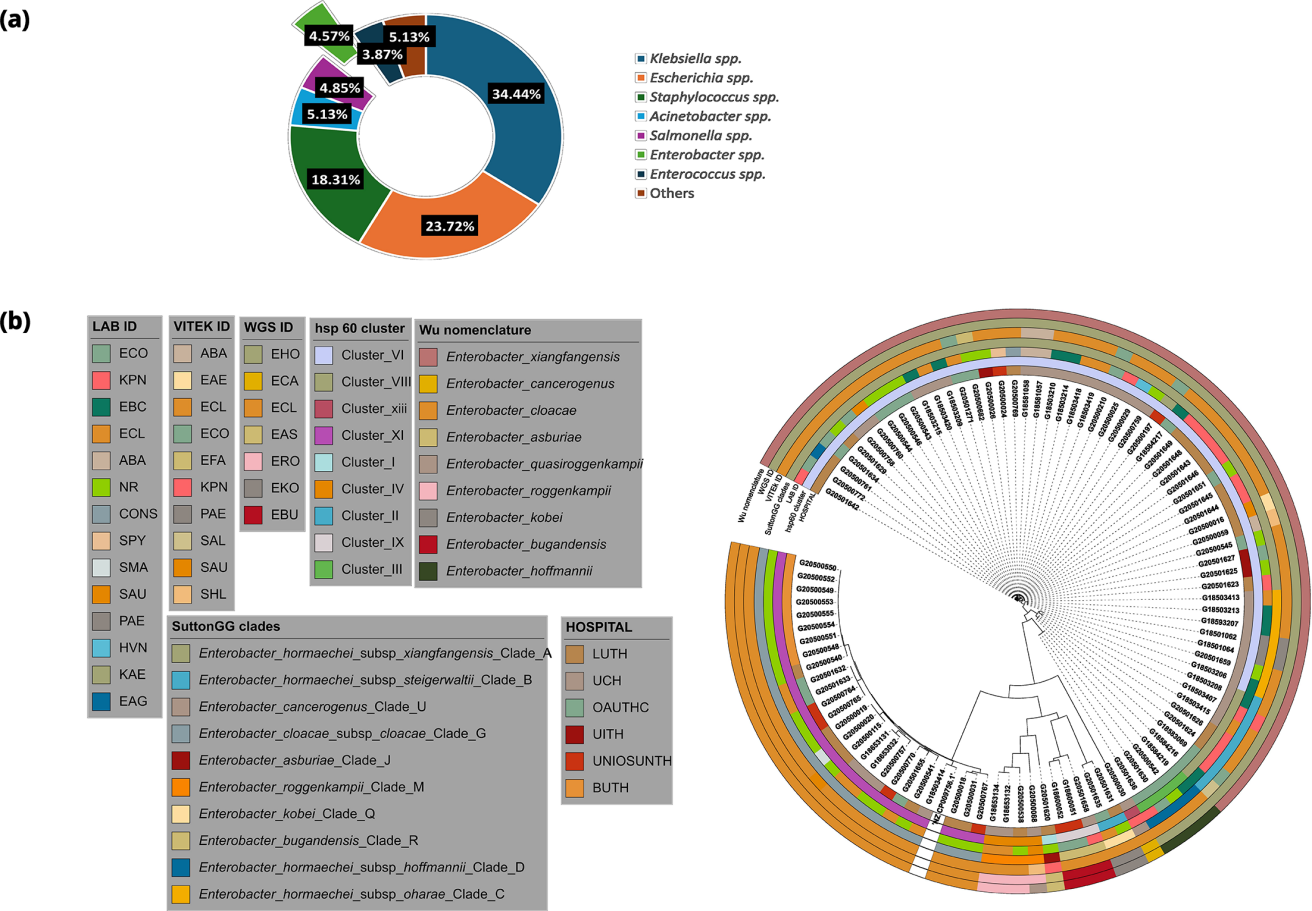
(a): Proportion of genera received from invasive infections from six sentinels in the Nigerian AMR surveillance system (2014–2020). (b) Maximum likelihood tree showing whole-genome sequence identifications of *Enterobacter* isolates recovered from patients admitted to six hospitals in southwestern Nigeria, juxtaposed with identifications of *Enterobacter* species by the diagnostic lab sentinels and reference lab VITEK 2 reidentification, their clades and Hoffman clusters.

Sentinel labs sent a total of 63 isolates as *Enterobacter* spp., and of these, 27 were verified as *Enterobacter* by VITEK 2, out of which WGS eventually identified 13 as belonging to the genus. An additional 85 isolates were sent as *Enterobacteriaceae*, as species belonging to other families, or as unidentified, and they were subsequently identified by WGS as *Enterobacter* (Fig. 1b). In total, 98 (4.57%) *Enterobacter* isolates from 2014 to 2020 were received at the national reference laboratory, representing the sixth most common genus isolated in bloodstream infections (after *Klebsiella*, *Escherichia*, *Staphylococcus*, *Acinetobacter* and *Salmonella*) (Fig. 1a). Of these 98 isolates, 61 (62.25%) were identified by WGS as *E. hormaechei*, 26 (26.53%) as *E. cloacae*, and the rest, 11 (11.22%) were identified as *Enterobacter roggenkampii* (4), *Enterobacter bugandensis* (3), *E. kobei* (2), *E. asburiae* (1) and *Enterobacter cancerogenus* (1) (Fig. 1b). The basic information of the 98 WGS *Enterobacter* strains, including their genome sizes, GC contents and genome coverage, is shown in Table S2, available in the online Supplementary Material.

**Fig. 2.**
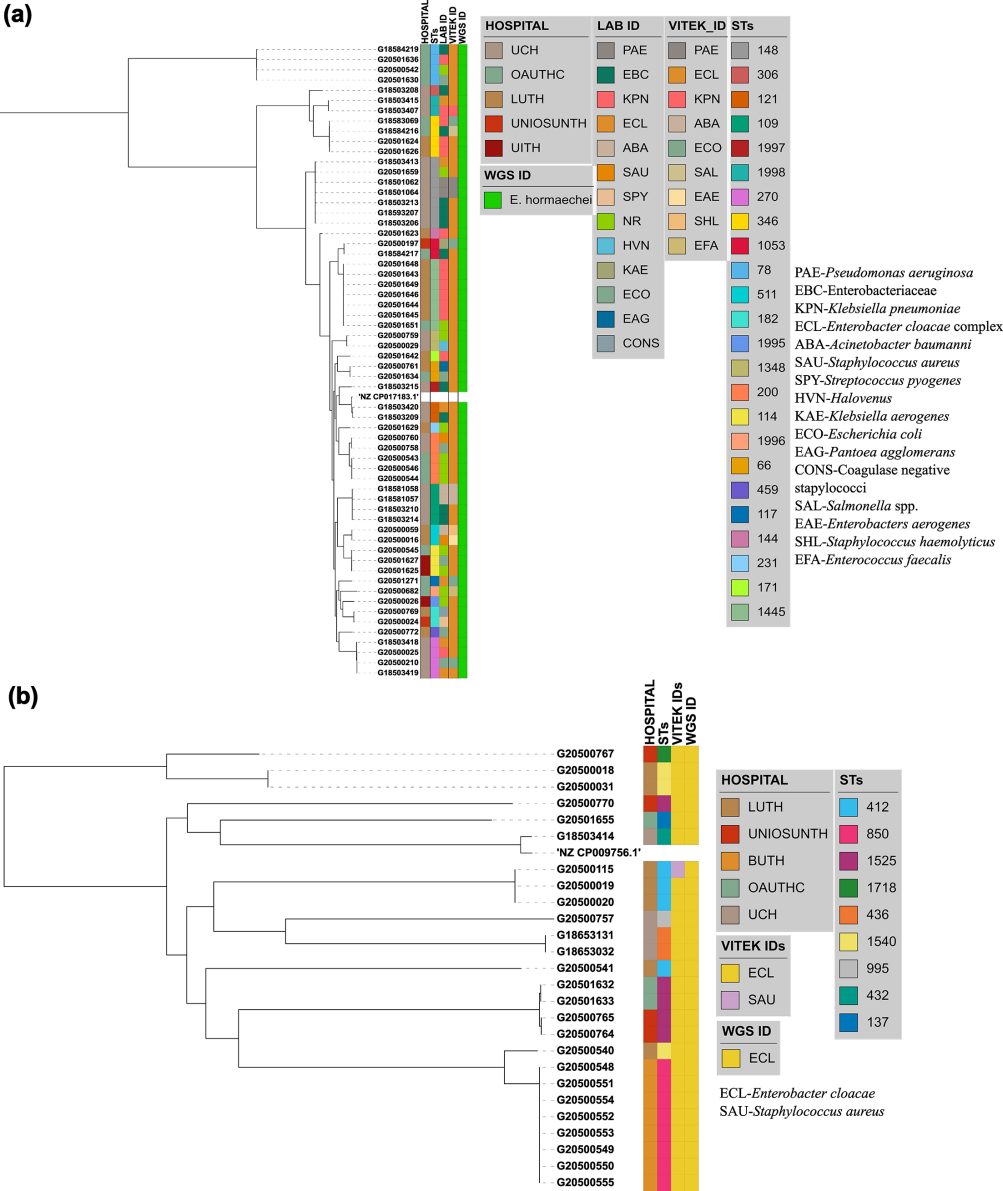
Initial identification of the two most frequently detected *Enterobacter* species by reference and sentinel laboratories, whole-genome sequence ID and their STs (**a**) *E. hormaechei* (**b**) *E. cloacae*

Forty-nine of the 61 WGS-identified *E. hormaechei* isolates were identified as *Klebsiella pneumoniae* (*n*=14, 29%), *Enterobacteriaceae* (*n*=11, 22%), ECC (*n*=6, 12%), *Escherichia coli* (*n*=6, 2%), *Acinetobacter baumannii* (*n*=3, 6%), *Staphylococcus aureus* (*n*=2, 4%), *Pseudomonas aeruginosa* (*n*=3, 6%), *Pantoea agglomerans* (*n*=1, 2%), coagulase-negative *Staphylococcus* (*n*=1, 2%), *Streptococcus pyogenes* (*n*=1, 2%) and *Halovenus* (*n*=1, 2%) at the sentinel laboratories (Fig. 2a).

At the national reference laboratory, the 61 WGS-identified *E. hormaechei* were initially identified (using VITEK 2) as *Enterobacter cloacae* complex (*n*=48, 79%), *E. coli* (*n*=4, 7%), *A. baumannii* (*n*=2, 3.3%), *E. aerogenes* (*n*=1, 1.6%), *Salmonella spp*. (*n*=1, 1.6%), *P. aeruginosa* (*n*=1, 1.6%) and *K. pneumoniae* (*n*=1, 1.6%). The selection of Gram-positive VITEK cards after Gram miscalling resulted in the misidentification of three further isolates as enterococci or staphylococci: *Enterococcus faecalis* (*n*=1), 1.6%, *Staphylococcus haemolyticus* (*n*=1, 1.6%) and *S. aureus* (*n*=1, 1.6%) (Fig. 2a and Table S3). VITEK 2 percentage probabilities of identification of the species are shown in Table S3. The proportion of *E. cloacae* (the second most abundant species identified among the *Enterobacter* spp.) identified biochemically, using VITEK 2 as *E. cloacae* was 96.1% (*n*=25), while 3.9% (*n*=1) was identified as *S. aureus*. The sensitivity, specificity, positive predictive and negative predictive values (100%, 0%, 100% and 0%, respectively) [27,28] for *E. cloacae* complex identification in this study by VITEK 2 using GN ID cards (21341) show that this method is adequate for *E. cloacae* but suboptimal for *E. hormaechei* (0% for all four values) in our setting (Table S4).

Altogether, retrospective (2017 and prior) and prospective (2014–2020) *Enterobacter* isolates were received from six tertiary hospitals: University College Hospital (UCH), Ibadan (*n*=35); Lagos University Teaching Hospital (LUTH), Lagos (*n*=30); Obafemi Awolowo University Teaching Hospital (OAUTHC), Ile-Ife (*n*=18); Babcock University Teaching Hospital (BUTH), Ogun (*n*=8); Osun State University Teaching Hospital (UNIOSUNTH), Osogbo (*n*=4); and University of Ilorin Teaching Hospital (UITH), Ilorin (*n*=3) (Fig. 6b, c).

*Enterobacter hormaechei* strains – the most abundant *Enterobacter* species identified – were detected in 5 of the 6 sentinels and were quite diverse (Simpson’s diversity index=0.937) as they belonged to 22 different STs (Fig. 2a), including the novel STs, ST1995 (*n*=1), ST1996 (*n*=1), ST1997 (*n*=1) and ST1998 (*n*=2). This species was most commonly identified from UCH, representing 25/35 of the *Enterobacter* species (Fig. 2a) and encompassing 7 STs, including two novel STs – ST1997 and ST1998. *E. hormaechei* were also retrieved from the sentinel sites in Ile-Ife (OAUTHC, 15 strains belonging to 9 STs, including a novel ST-ST1996), Lagos (LUTH, 16 strains belonging to 9 STs), Ilorin (UITH, 3 strains belonging to STs 114 and novel ST1995) and Osogbo (UNIOSUNTH, 2 strains belonging to STs 1053 and 182) (Fig. 2a). No particular STs were seen to be shared across the hospitals. The STs 148 and 1445 were the most prevalent among the isolates.

*E. cloacae* (*n*=26) were collected from UCH (*n*=4), LUTH (*n*=7), UNIOSUNTH (*n*=4), BUTH (*n*=8) and OAUTHC (*n*=3). Isolates belonged to 10 different STs: 432 (1), 436 (1), 1540 (2), 412 (3), 850 (9), 760 (1), 995 (1), 1525 (5), 1718 (2) and 137 (1), with ST850 being the most prevalent. Three STs (ST432, ST436 and ST995) were detected among four strains from UCH, four STs (ST1540, ST412, ST760 and ST850) in seven strains from LUTH, two STs (ST1718 and ST1525) in four strains from UNIOSUNTH, ST850 in eight strains from BUTH, and ST137 and ST1525 in three strains from OAUTHC. *E. cloacae* ST850 was commonly found in BUTH and LUTH (Fig. 2b).

### Antimicrobial resistance genes and phylogenetic relationships among *Enterobacter* isolates

**Fig. 3.**
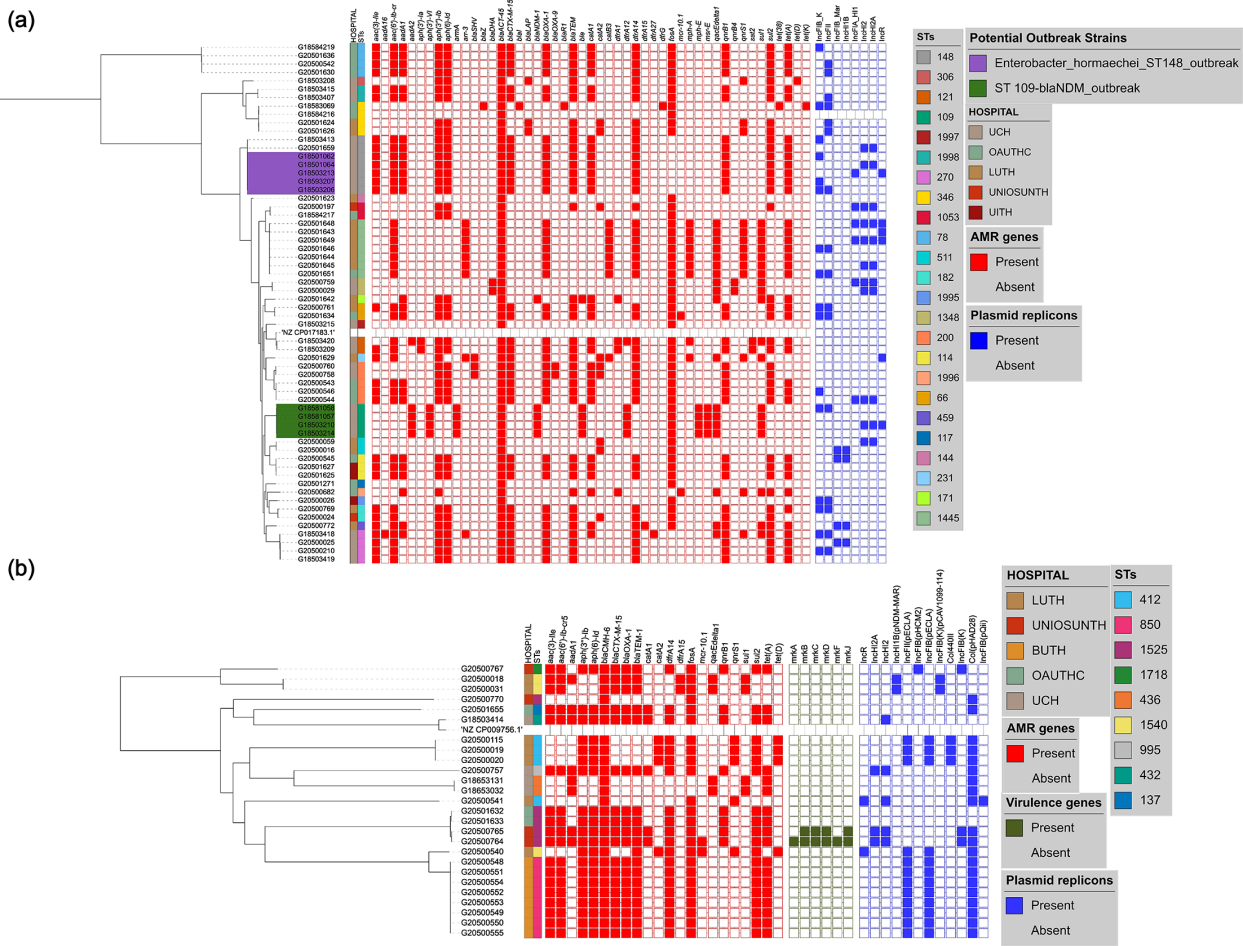
Maximum likelihood phylogenetic trees showing the relationship among *Enterobacter* isolates belonging to the most frequently encountered species from different hospitals, STs, AMR genes and plasmid replicons detected. (**a**) *E. hormaechei* (**b**) *E. cloacae.*

Carbapenems, beta-lactams, fluoroquinolones, cephalosporins, aminoglycosides and colistin are antibiotics commonly used to treat infections caused by *Enterobacter* species [29,30]. Genes conferring resistance to these antibiotics – carbapenems (*bla*_NDM_, 5), beta-lactams/cephalosporins (*bla*_ACT-45_, 61; *bla*_TEM_, 39; *bla*_CTX-M-15_, 33; *bla*_OXA_, 40; *bla_SHV_*, 3; *bla_DHA_*, 2), fluoroquinolones [*aac(6′)-Ib-cr*, 38; *qnrB1*, 32], fosfomycin (*fosA*, 49), chloramphenicol (*catA1*, 29; *catA2*, 9; *catB3*, 8), macrolide (*mphA*, 9; *mphE*, 4; *msrE*, 4), sulphonamide (*sul1*, 19; *sul2*, 38), tetracycline [*tet(38*), 1; *tet(A*), 38; *tet(D*), 1; *tet(K*), 1], quinolone (*qnrB1*, 32; *qnrB4*, 2; *qnrS1*, 13), trimethoprim (*dfrA1*, 4; *dfrA12*, 5; *dfrA14*, 44; *dfrA15*, 1; dfrA27, 1; *dfrG*, 1), quaternary ammonium compounds (*qacEdelta1*, 18), aminoglycosides [*aadA1*, 25; *aadA2*, 5; *aph(3′)-Ia*, 2; *armA*, 4; *aac (3)-Ile*, 29; *aph(3′)-Ib*, 40; *aph(6)-Id*, 38), and colistin (*mcr10.1*, 2) – were detected in *E. hormaechei* genomes (*n*=61) (Fig. 3a). Fifty-six strains were classified as multidrug-resistant (as defined by [18,18]) due to the *in silico* detection of more than two of these genes.

*Enterobacter* spp. are known to carry core chromosomal AmpC-type beta-lactamases and their variants [31] (Table S5). While various other *bla*_ACT_ alleles have been associated with *E. hormaechei* in the literature [31,32], by using ResFinder to analyse the resistance genes, we found that all 61 isolates in this study carried *bla*_ACT-45_. This gene was earlier reported to occur naturally in *E. hormaechei* subsp. *xiangfangensis* and contribute to antibiotic resistance mechanisms observed in these strains [33]. Meanwhile, further analysis of the *ampC* variants using AMRFinderPlus and CARD revealed 27 different *ampC* variants in the 98 isolates in this study (Table S5). The most common variant in this study was bla_ACT-16_ and was found to be associated with *E. hormaechei*. Of the 98 *Enterobacter* strains we sequenced, all but 16 isolates carried, in addition to core AmpC-type beta-lactamase-encoding genes, one or more acquired beta-lactamase genes associated with mobile elements, as well as other resistance genes that confer resistance to the aminoglycosides, trimethoprim, colistin, fluoroquinolone, fosfomycin, chloramphenicol, macrolide, sulphonamide and tetracycline (Fig. 3a). Thirty-three of the *E. hormachei* and 18 *E. cloacae* carried *bla*_CTX-M-15_. In 12/51 of these cases, an IncFIb plasmid replicon was also detected. IncFIA_HI1, IncHI2, IncHI2A and IncR plasmid replicons were also detected among the *bla_CTX-M-15_*-carrying strains. Carbapenemase gene *bla*_NDM-1_ was found in five of the *E. hormaechei* genomes. *dfrA* alleles conferring trimethoprim resistance were almost ubiquitous, with *dfrA14* predominating in 51/61 and 22/26 *E. hormaechei* and *E. cloacae* genomes, respectively.

The plasmid-borne resistance *mcr*10.1 gene was found in two *hormaechei* strains. The *mcr10.1*-carrying *hormaechei* strains belonged to ST 66 and novel ST 1996 and had no plasmid replicon type in common, according to the output from plasmid finder. The phylogeny (Fig. 3a) shows that isolates from the same location cluster together, often carrying identical resistance genes, suggesting very local epidemiologies for *E. hormaechei* lineages and that the predominance of this species throughout the whole network is not due to clonal expansion of one or a few clones.

We observed that *E. cloacae* strains (Fig. 3b) exhibited multidrug resistance. Frequencies of resistance genes found in isolates (*n*=26) were, for aminoglycoside [*aac(3)-Ile*, 18; *aac(6′)-Ib-cr5*, 18; *aadA1,* 8; *aph(3′)-Ib*, 20; *aph(6)-Id*, 20], beta-lactams (*bla_CMH-6_*, 26; *bla_CTX-M-15_*, 18; *bla_OXA-1_*, 18; *bla_TEM-1_*, 19), chloramphenicol (*catA1*, 5; *catA2*, 4), sulphonamides (*sul1*, 4; *sul2*, 20), tetracycline (*tetA*, 18; *tetD*, 4), trimethoprim (*dfrA14*, 20; *dfrA15*, 2), fosfomycin (*fosA*, 24), quinolone (*qnrB1*, 8; *qnrS1*, 5) and colistin (*mcr-10.1*, 2). Plasmid-borne resistance gene *mcr10.1* was observed in two *E. cloacae* isolates belonging to ST1718 and ST850 isolated from UNIOSUNTH and LUTH, respectively (Fig. 3b). These strains had otherwise different resistance genes and plasmid replicon profiles.

We calculated the concordance between the phenotypic AMR (VITEK 2 AMR result) and the genotypic AMR (WGS AMR result) for the *E. cloacae* and *E. hormaechei* species (Table S6). A concordance of 1 signifies a 100% agreement between phenotypic and genotypic antibiotic resistance. For *E. hormaechei* resistance data, ampicillin, cefuroxime, cefuroxime axetil, imipenem, meropenem and amikacin showed 100% concordance. On the other hand, cefepime and cefoperazone/sulbactam showed less than 50% concordance, while the other antibiotics showed greater than 50% but less than 100% concordance. For *E. cloacae*, piperacillin/tazobactam, cefoperazone/sulbactam, amikacin and colistin showed poor concordance, reflecting that care needs to be taken in the choice of beta-lactam antimicrobials used for phenotypic testing, particularly in those cases.

### Potential *Enterobacter* healthcare-associated infection outbreaks detected

Two potential hospital outbreaks were retrospectively detected in this study among the *E. hormaechei* strains at the UCH facility. An ST109 cluster carrying the *bla*_NDM-1_ carbapenemase gene (Fig. 3a) was comprised of strains that were phenotypically sensitive to meropenem, imipenem and ertapenem with Minimum Inhibitory Concentrations (MICs) of ≤ 0.25, 1 and 0.5 and ≤0.5, respectively (Table S7). These isolates also did not demonstrate phenotypic resistance to other beta-lactams attributable to *bla*_ACT-45_. The SNP distance among ST109-bearing strains was between 0 and 1, while the range of SNP distance between these putative outbreak isolates and *E. hormaechei* that are not part of the outbreak is between 144 and 258 SNPs (Fig. S1). The ST109 putative outbreak strains carried aminoglycoside-resistance genes not seen in any of the other *E*. *hormaechei* – *aph(3′)-VI* and *armA*, as well as *aadA2*, *aph(3′)-Ia*, *bla*_ACT_, *bla*_NDM_, *bla*_TEM_, *ble*, *dfrA12*, *fosA*, *mphE*, *msrE* and *sul1* genes. Only one isolate outside this likely outbreak cluster carried the *blaNDM-1* gene. It belonged to ST1445, was from a different facility and contained a completely different repertoire of resistance genes (Fig. 3a). The outbreak strains carried resistance genes belonging to six antimicrobial classes: aminoglycoside, beta-lactamase, trimethoprim, carbapenem, macrolide and sulphonamide resistance, compared to the median number of resistance classes conferred by genes in non-ST109 strains (range, 3–5). All four genomes contained IncFIB_Mar and IncHIB plasmid replicons, which are not found in other isolates.

A second cluster of five ST148 strains, also from UCH, harboured IncFIB_K and IncFII plasmid replicons and carried genes conferring resistance to aminoglycosides [*aac(6′)-Ib-cr*, *aac (3)-Ile*, *aadA1*, *aph(3′)-Ib* and *aph(6)-Id*], beta-lactams (*bla*_ACT45_, *bla*_CTX-M-15_, *bla*_OXA-1_ and *bla*_TEM_), phenicol (*catA1*), quinolones (*qnrB1*), sulphonamides (*sul2*), trimethoprim (*dfrA14*), and tetracyclines (*tet*A) (Fig. 3a). SNP distance among strains within the ST148 cluster was between 0 and 1, while the range of SNP distance between these outbreak isolates and the other strains is between 27,542 and 31,513 (Fig. S1). They were phenotypically resistant to cephalosporins, carbapenems, aminoglycosides, quinolones and trimethoprim (Table S7).

Sentinel laboratories in Nigeria can request accelerated sequencing of suspected outbreak clusters [14]. However, although these likely outbreaks, for which retrospective time (other than year) and place information are not available, occurred at the sentinel that had the greatest success at identifying *Enterobacter* genus strains, both clusters contained isolates that were misclassified as different species at the sentinel and reference lab (VITEK 2) levels, which would have hampered WGS-independent cluster identification.

### Comparative genomics and phylogenomics of *Enterobacter* spp. in Nigeria

**Fig. 4.**
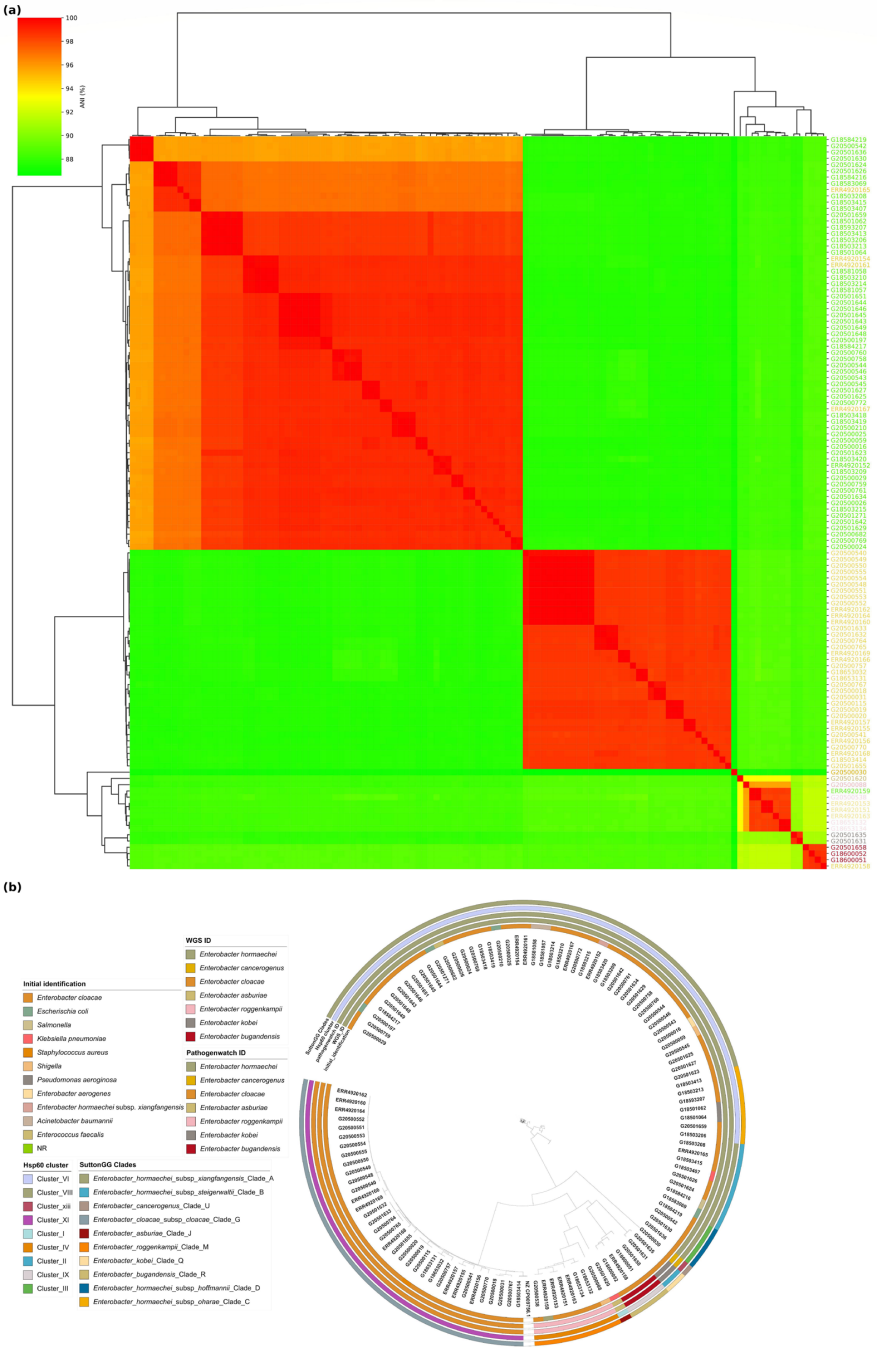
Comparison of genomes from this study and those of isolates from elsewhere in Nigeria. Genome IDs are prefixed by ERR* if submitted by Sands *et al*. [8] or G* (this study). (**a**) ANI analysis of genome-sequenced *Enterobacter* spp.: *E. hormaechei* (EHO), *E. cloacae* (ECL), *E. bugandensis* (EBU), *E. asburiae* (EAS), *E. roggenkampii* (ERO) and *E. kobei* (EKO). (**b**) Maximum likelihood tree illustrating the phylogeny among all available Nigerian genomes in the ENA.

To enable us to analyse the distribution of *Enterobacter* lineages nationally, we downloaded all *Enterobacter* genomes associated with Nigeria that can be retrieved from the ENA under project number PRJEB33565. All the genomes not from the current study (see Table S1) arose from the study by Sands *et al*., a rigorous WGS-based neonatal sepsis study, and were submitted as *Enterobacter* species (19) – specifically, *cloacae* (17), *hormaechei* subsp. *xiangfangensis* (1) and *hormaechei* (1). These were included in our ANI analysis. We compared the strains from Sands *et al*. [8] and this study and found that four *E. cloacae* genomes clustered with *E. hormaechei*, three with *E. roggenkampii*, one with *E. bugandensis* and the others with *E. cloacae*, respectively. An *E. hormaechei* genome from Sands *et al.* [8] clustered with *E*. *roggenkampii* and the other with *E*. *hormaechei* from this study (Fig. 4a). We re-identified the Sands *et al.* [8] *Enterobacter* isolates using our assembly and speciation pipelines. Sands *et al. E. cloacae* were identified as *E. roggenkampii* (3), *E. hormaechei* (4), *E. bugandensis* (1) and *E. cloacae* (9), the *E. hormaechei* subsp. *xiangfangensis* as *E. hormaechei* and the *E. hormaechei* as *E. roggenkampii*. Sands *et al.* [8] used both blast and Pathogenwatch to identify their bacterial species. The identities from Pathogenwatch of Sands *et al*. [8] genomes and the genomes from our study were finally correlated with the identities from our pipelines. The output from Hoffman’s classification yielded identities aligned with our pipeline’s results (Fig. 4b).

**Fig. 5.**
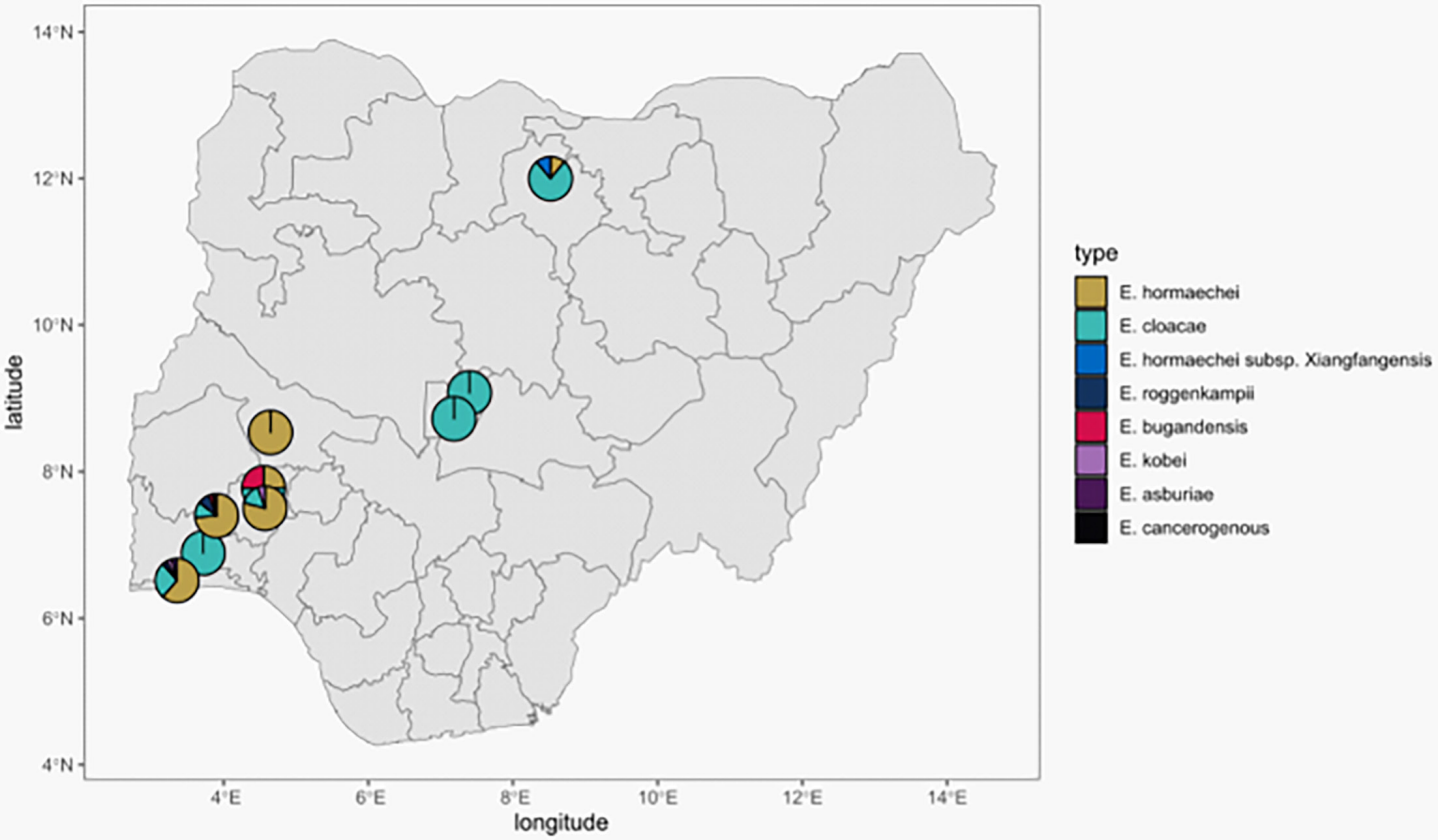
Geographic source of genome-sequenced *Enterobacter* in Nigeria. Isolates originated from southwestern Nigeria (this study) and north and central Nigeria [8].

The Sands *et al*. [8] isolates were from northern Nigeria [National Hospital, Abuja (NN), Wuse District Hospital, Abuja (NW), and Murtala Muhammad Specialist Hospital, Kano (NK) in Nigeria], which were geographically distinct from the area where our isolates were collected. Altogether, the two studies identified 43 *Enterobacter* STs, 11 of which were found at more than one facility. Additionally, 2 (STs 109 and 850) were reported in this study and the Sands *et al.* [8] study. Our data contain no clinical or outcome information on the isolates. However, Sands *et al.* [8] found that the STs 1238, 850, 103 and 544 were commonly associated with fatal infections. This study recovered isolates belonging to these STs from neonatal sepsis infections. The identities from all nine hospitals are shown on the Nigerian map (Fig. 5).

A comparative genomic analysis of the 98 *Enterobacter* strains was carried out. In this study, we observed that the pangenome of *Enterobacter* strains is open. *E. cloacae* has a more conserved genome and less variation in gene content across strains than *E. hormaechei*, which exhibits greater diversity (Fig. S2A, S2B). In *E. cloacae*, 7,615 genes were identified, comprising 3,490 core genes and 1,128 unique genes. In contrast, *E. hormaechei* exhibited a significantly larger pangenome, with 12,521 total genes, including 3,326 core and 4,037 unique genes. COG and KEGG analysis showed that the pangenome is also characterised by a high proportion of genes associated with carbohydrate metabolism, amino acid metabolism, energy metabolism, membrane transport and signal transduction.

## Discussion

In this study, we performed whole-genome sequencing of bloodstream isolates submitted to the Nigerian surveillance system. Bloodstream isolates collected between 2014 and 2020 included *S. aureus*, *E. coli*, *K. pneumoniae*, *A. baumannii*, *Salmonella*, *P. aeruginosa* and *Enterobacter* spp. as the most common genera (Fig. 1). Of 2,360 isolates received, 63 were initially sent as presumptive *Enterobacter*, and 98 (4.2%) were eventually identified as *Enterobacter* species. Currently, there are 45 (22 named and 21 without assigned names) species of *Enterobacter* [1,31, 34], and 7 were identified in this study (Fig. 6b). Among the 22 named *Enterobacter* spp., seven belong to the ECC, and they include *E. cloacae*, *E. hormaechei*, *Enterobacter mori*, *E. asburiae*, *E. ludwigii*, *E. nimipressuralis* and *E. kobei*. All but *E. mori*, *E. ludwigii* and *E. nimipressuralis* were identified in this study. We also identified *E. roggenkampii*, *E. bugandensis* and *E. cancerogenus*, which are not members of the *cloacae* complex (Fig. 6b).

*E. hormaechei* is frequently encountered in clinical specimens and is commonly considered a nosocomial pathogen [1]. However, there are only a few reports of bloodstream infections caused by *E. hormaechei* in Africa. Duru *et al.* [10] reported the identification of *Enterobacter* spp. from blood samples; however, the taxonomic resolution was limited to the genus level. In contrast to our study, which identified *E. hormaechei* as the most common *Enterobacter* species, a previous study conducted more than a decade ago [12] in Benin City, Nigeria, identified *Enterobacter sakazakii* and *E. aerogenes* as the most prevalent *Enterobacter* species from clinical samples, which included blood and did not report *E. hormaechei*. In the Sands *et al*. [8] study, *Enterobacter* was prominent in Nigeria. The conspicuous dearth of any previous report on *E. hormaechei* from other clinical samples in Nigeria is likely due to the misidentification of *Enterobacter* species using biochemical identification methods.

Identifying the *Enterobacter* genus is often challenging [1]. The genus is often inaccurately identified by clinical laboratories using biochemical and other phenotype-based tests, VITEK 2 and MALDI-TOF mass spectrometry [5,35]. Resource-limited settings may face heightened challenges with identifying bacterial pathogens; therefore, supportive reference laboratory services are critical [6,14, 36]. In this study, four *Enterobacter* species were submitted as Gram-positive strains, and three of these belonged to the most common species – *E. hormaechei*. Isolates were submitted after identification by tube- or strip biochemical test-using labs as *Enterobacteriaceae* (*n*=11) or *E. cloacae* (*n*=5) or were misidentified by sentinels as *A. baumannii* (*n*=3), *Pseudomonas* (*n*=2), *K. pneumoniae* (*n*=14), *E. coli* (*n*=6), *Halovenus* (*n*=1), *Klebsiella aerogenes* (*n*=2). At the reference laboratory, VITEK 2 lacked the resolution to delineate these species as *E. hormaechei*, although it did place most of them in the ECC. In this study, application of WGS has enabled the identification of these organisms accurately at the species and subspecies level and revealed that *E. hormaechei* was commonly isolated in all participating hospitals, except for BUTH (from which, overall, only a few isolates were obtained) (Figs2b  6b). Within-species misclassifications have little consequence for patient management but prevent early identification of clusters, which is crucial for infection prevention and control. Our results and the important sub-specific nuances we found emphasize the need to integrate WGS into routine clinical diagnosis of infectious diseases. Also, there is a need to update the VITEK 2 and MALDI-TOF databases to improve the accuracy of speciation.

Analysis of the *hsp60* gene (a housekeeping gene) has conventionally been used to sub-classify the ECC into 13 genetic clusters (Hoffman clusters I–XII and an unstable sequence crowd xiii) [37]. A whole-genome analysis study (1,997 *Enterobacter* genomes) updated the taxonomy of the *Enterobacter* genus [9]. ANI thresholds between 94 and 96.5% and 97–98% for subspecies have good correlations with current species designations [38]. ANI was used for classifying strains in the ECC into 22 clades (A–V), which correspond to Hoffman clusters (I–XII) [39] (Figs 6b and 4b). Making connections across the *Enterobacter* literature is challenging because of the different schemes to which multi-locus sequence typing, offering much finer sub-classification, has been added. WGS approaches make it possible to classify strains according to all schemes easily and, therefore, compare disparate datasets. This enabled us to collate information from the Sands *et al*. study, which was conducted in various locations in Nigeria over an overlapping timescale. Like Sands *et al*. [8], we found multi-locus sequence typing helpful for understanding population structure.

Comparative genomics, especially pan-genome analysis, provides a more accurate picture of what a bacterial species truly is [40]. The pangenome analysis performed in this study shows that *E. hormaechei* is more genetically diverse than *E. cloacae*, and both have open pangenomes. This study also reveals that the *Enterobacter* pangenome is open, particularly for *E. hormaechei*, indicating ongoing gene acquisition and high genomic plasticity. The open nature of the pangenome suggests that *Enterobacter* spp., especially *E. hormaechei*, possess a high capacity for horizontal gene transfer and niche adaptation. The stark difference in genes present in *E. hormaechei* compared to *E. cloacae* highlights the greater genetic diversity and dynamic evolution of *E. hormaechei* compared to *E. cloacae*. The continual increase in gene families with additional genomes reflects the open nature of their pangenomes, characterized by a conserved core and a variable accessory gene pool. This aligns with other studies that have investigated the *Enterobacter* spp. pangenome [41,44]. Moreover, the lower variation in gene content of *E. cloacae* suggests that it may occupy more similar niches or undergo fewer horizontal gene transfers than *E. hormaechei*. COG and KEGG analyses suggest that *Enterobacter* strains can utilize diverse carbon and nitrogen sources and adapt to nutrient-limited environments. In both *E. hormaechei* and *E. cloacae*, the pangenome showed considerable expansion in genes associated with transcription, DNA replication, recombination and repair. The pangenome’s dynamic and continuously evolving nature reflects its ability to thrive in various ecological niches and acquire new traits via horizontal gene transfer [45,46]. This could also account for these strains’ high resistance traits and virulent potential.

Our isolates belonged to 22 different STs, including novel STs (now assigned ST1995, ST1996, ST1997 and ST1998), which, with the 12 STs reported by Sands *et al*. (of which 2 STs were seen in both studies), show that *E. hormaechei* populations circulating within Nigerian hospitals are considerably diverse. *E. hormaechei* strains were multidrug resistant with the detection of aminoglycoside, cephalosporin, chloramphenicol, macrolide, colistin and carbapenem resistance genes. *bla*_NDM_*-*harbourin*g* ST109 and ST148 (Fig. 3a) appear to represent two different outbreaks comprised of isolates with SNP distances of ≤1 and ≤1, respectively. ST148 *Enterobacter* strains have been known to cause outbreaks in the past and have been identified among species isolated at hospitals. A study in Canada that investigated carbapenemase-producing *Enterobacterales* transmission clusters at a hospital system identified *bla*_VIM-1_-positive ST148 strains harbouring plasmid replicons IncR, HI2 and HI2A [47]. The ST148 strains in this study did not carry any carbapenemase gene but were multidrug-resistant (Fig. 3a). They were phenotypically resistant to imipenem and meropenem, with MICs of ≥8 and ≥16, respectively (Table S7). This phenotype may result from overexpression of the chromosomal *ampC* gene alongside alteration in outer membrane transcriptome balance, which is known to proffer other phenotypes such as carbapenem resistance [48]. OXA-48-like-producing ST109 *E. cloacae* was implicated alongside 22 *K. pneumoniae* and 3 *E. coli* in outbreaks of OXA-48-like-producing *Enterobacteriaceae* in Czech hospitals in 2015. The ST109 *E. cloacae* strain harboured, in addition to the *bla*_OXA-48_ gene, *bla*_CTX-M-15_, *bla*_OXA-1_ and *bla*_TEM-1_ [49]. The ST109 strains in this study carried only the core *bla*_ACT-45_ and *bla*_NDM-1_ beta-lactamase genes (Fig. 3).

*E. cloacae* was identified in all the hospitals, excluding UITH. A total of 26 isolates belonged to 10 different STs, with ST850 being the most prevalent, with SNP differences of ≤2. Nigeria was the only country from which Sands *et al.* [8] recovered *Enterobacter* spp. from every sentinel, similar to our study. The ST850 *E. cloacae* strains from our study are not very distantly related to the ST850 *E. cloacae* genomes from Sands *et al*., with an SNP distance range of between 70 and 89. Altogether, they identified three ST850 *E. cloacae* among antimicrobial-resistant Gram-negative bacteria that cause neonatal sepsis in seven low- and middle-income countries, and all were reported from Nigeria. Unlike our study, Sands *et al*. sentinels were in northern Nigeria. Thus, while our ST850 isolates appear to be focused on one sentinel, this clade may be circulating widely in Nigeria. *E. cloacae* isolates in this study were resistant to aminoglycosides, cephalosporins and colistin. The presence of the plasmid-borne colistin resistance gene, *mcr-10.1,* in two *E. cloacae* strains in this study is very worrisome, as colistin, which is difficult to access in Nigeria, is one of the last available antibiotics used in the treatment of carbapenem-resistant infections. Moreover, *mcr* genes are easily transmitted. Although there are few reports of colistin-resistant *Enterobacter* in Africa, colistin-resistant *E. cloacae* was recently identified in Sierra Leone. The strain belonged to ST850 and was resistant to cefazolin, gentamicin and trimethoprim [50]. ST850 strains from this study were colistin-sensitive, and the colistin-resistant *E. cloacae* strains belonged to ST1525 and ST760. They also possessed *bla*_CTX-M-15_, *bla*_TEM1_, *bla*_CMH_, *qnrS*, *qnrB*, *tetD*, *aph(6)-id*, *aadA1*, *dfrA*, *catA2* and *sul2* genes, which confer resistance to beta-lactams, quinolone, tetracycline, trimethoprim, chloramphenicol, aminoglycoside and sulphonamides.

The importance of *Enterobacter* species as bloodstream pathogens in Nigeria has, heretofore, been overlooked because precise identification of this genus poses a challenge for clinical laboratories due to limited biochemical capacity and the complicated taxonomy of the genus. In this study, WGS enabled the accurate delineation of members of this genus, revealing *E. hormaechei* as the predominant species. It also allowed for retrospective identification of earlier missed outbreaks. In this study, the retrospective identification of potential outbreaks and the detection of genes conferring resistance to last-line drugs, including carbapenems and colistin, is concerning.

The detection of *E. hormaechei* as the most prevalent *Enterobacter* bloodstream isolate, along with the identification of key *E. cloacae* lineages in this study, underscores the need to enhance clinical laboratory identification and maintain ongoing surveillance of this genus. This study emphasizes the importance of WGS in bacteriology, but it also demonstrates that concentrating WGS resources at the reference laboratory is a barrier to identifying lineages and clusters that are important at the patient care level, which needs to be addressed in our setting in the future.

## Supplementary material

10.1099/mgen.0.001508 Uncited Supplementary Material 1.

10.1099/mgen.0.001508 Uncited Table S1.

## Abbreviations

AMR: antimicrobial resistance
ANI: average nucleotide identity
BUTH: Babcock University Teaching Hospital
CARD: Comprehensive Antimicrobial Resistance Database
COG: Clusters of Orthologous Groups
ECC: *Enterobacter cloacae* complex
ENA: European Nucleotide Archive
GHRU: Global Health Research Unit
KEGG: Kyoto Encyclopedia of Genes and Genomes
LUTH: Lagos University Teaching Hospital
OAUTHC: Obafemi Awolowo University Teaching Complex
STs: sequence types
UCH: University College Hospital
UITH: University of Ilorin Teaching Hospital
UNIOSUNTH: Osun State University Teaching Hospital
WGS: whole-genome sequencing
