# Supplementary material for: Whole-genome sequencing reveals Enterobacter hormaechei as a key bloodstream pathogen in six tertiary care hospitals in southwestern Nigeria
Source: Microb Genom. 2025 Oct 6;11(10):001508. doi: 10.1099/mgen.0.001508 (PMC12500395; doi:10.1099/mgen.0.001508)
Supplement: Uncited Supplementary Material 1. [file mgen-11-01508-s001.pdf]

Figure S1: SNP distances for ST109 and ST148 strains with likely outbreak strains in darker shade

ST109-ndm	G20500059	G18581057	G18503214	G18503210	G18581058	G18503419
G20500016	0	27725	27703	27748	27676	31068
G20500059	0	27578	27561	27608	27542	31035
G18581057	27578	0	0	0	1	31383
G18503214	27561	0	0	0	1	31396
G18503210	27608	0	0	0	1	31432
G18581058	27542	1	1	1	0	31376
G18503419	31035	31383	31396	31432	31376	0
G20500210	31161	31461	31477	31513	31452	0

ST148	G18503206	G18593207	G18503213	G18501064	G18501062	G20501659	G18503413
G18503206	0	0	0	0	1	174	143
G18593207	0	0	0	0	1	170	144
G18503213	0	0	0	0	1	170	144
G18501064	0	0	0	0	1	170	144
G18501062	1	1	1	1	0	164	145
G20501659	174	170	170	170	164	0	254
G18503413	143	144	144	144	145	254	0

Figure S2: Pangenome matrix of (A) *Enterobacter hormaechei and* (B) *Enterobacter cloacae*

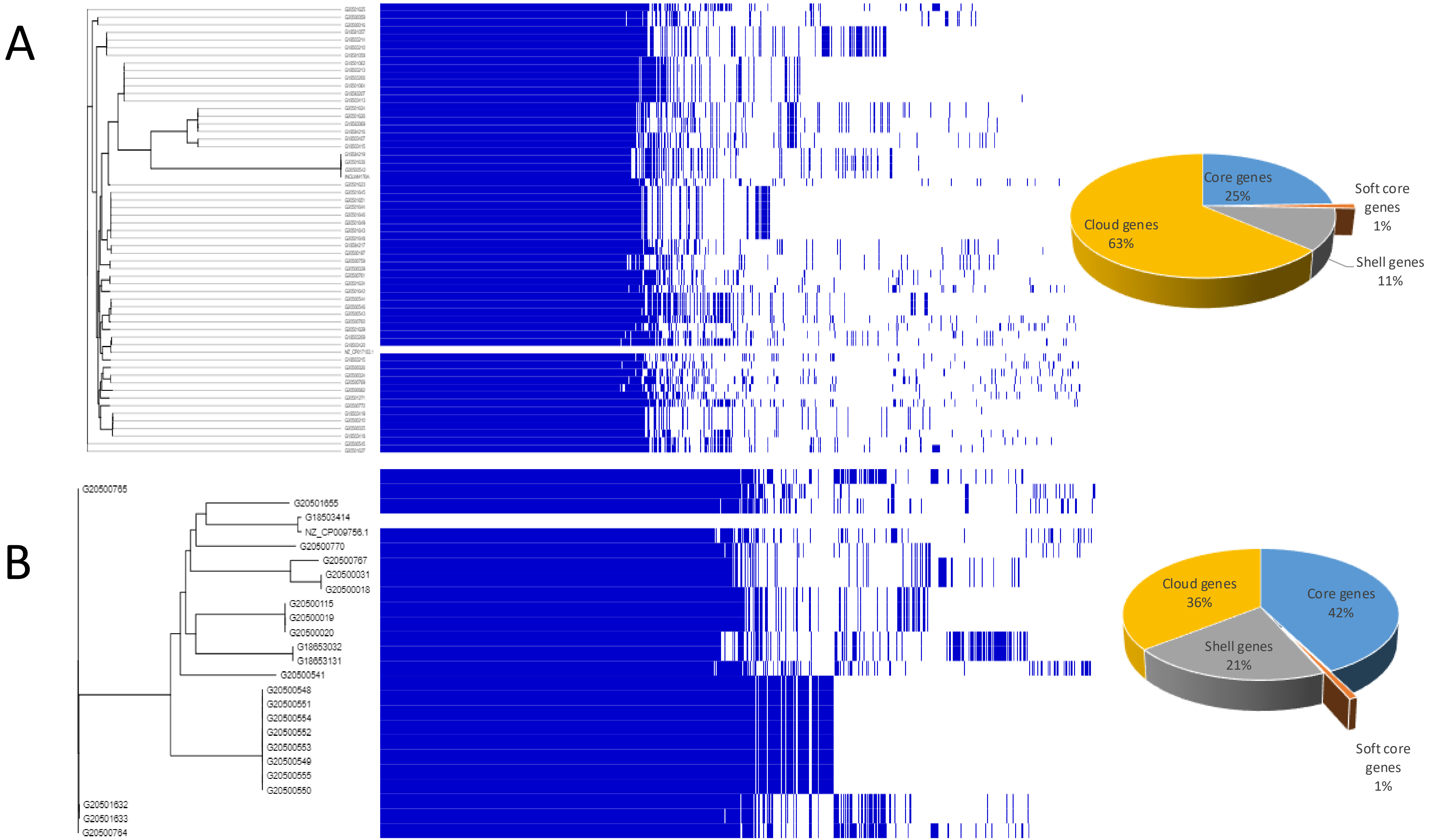

Table S1: a spreadsheet containing the sample accession numbers of Enterobacter isolates from this study

Table S2: Genomics characteristics of the 98 *Enterobacter* strains analysed in this study

Genome ID	Species	Number of contigs	Genome size (bp)	G+C_%
G18501062	*E. hormaechei*	64	4746118	55.5
G18501064	*E. hormaechei*	81	4832938	55.4
G18503206	*E. hormaechei*	98	4845587	55.4
G18503209	*E. hormaechei*	328	5176650	54.8
G18503210	*E. hormaechei*	45	5029476	54.9
G18503213	*E. hormaechei*	88	4836300	55.4
G18503214	*E. hormaechei*	54	5036258	54.8
G18503215	*E. hormaechei*	32	4826649	55.3
G18503407	*E. hormaechei*	39	4754749	55.5
G18503413	*E. hormaechei*	82	4821219	55.4
G18503415	*E. hormaechei*	49	4761565	55.5
G18503418	*E. hormaechei*	59	4823871	54.9
G18503419	*E. hormaechei*	52	4573626	55.4
G18503420	*E. hormaechei*	102	5258856	54.6
G18581057	*E. hormaechei*	55	5036376	54.8
G18581058	*E. hormaechei*	47	5028836	54.9
G18583069	*E. hormaechei*	153	7457990	47.4
G18584216	*E. hormaechei*	43	4746545	55.7
G18584217	*E. hormaechei*	37	4665014	55.5
G18584219	*E. hormaechei*	57	4916131	54.9
G18593207	*E. hormaechei*	83	4834009	55.4
G20500016	*E. hormaechei*	16	4619655	55.4
G20500024	*E. hormaechei*	64	4712547	54.9
G20500025	*E. hormaechei*	44	4575999	55.4
G20500026	*E. hormaechei*	23	4633810	55.6
G20500029	*E. hormaechei*	45	4513791	55.5
G20500059	*E. hormaechei*	18	4619027	55.4
G20500197	*E. hormaechei*	45	4792523	55.4
G20500210	*E. hormaechei*	43	4575851	55.4
G20500542	*E. hormaechei*	45	4923046	54.9
G20500543	*E. hormaechei*	52	5111370	54.8
G20500544	*E. hormaechei*	54	5111400	54.8
G20500545	*E. hormaechei*	54	4948422	54.9
G20500546	*E. hormaechei*	54	5111532	54.8
G20500682	*E. hormaechei*	51	4680717	55.6
G20500759	*E. hormaechei*	44	4513949	55.5

G20500760	*E. hormaechei*	115	5245840	54.8
G20500761	*E. hormaechei*	65	4849348	55.3
G20500769	*E. hormaechei*	55	4957550	55.2
G20500772	*E. hormaechei*	81	5141939	54.8
G20501271	*E. hormaechei*	25	4831087	55.3
G20501623	*E. hormaechei*	26	4623391	55.5
G20501624	*E. hormaechei*	55	4821819	55.7
G20501625	*E. hormaechei*	51	5010877	54.9
G20501626	*E. hormaechei*	60	4823647	55.7
G20501627	*E. hormaechei*	56	5059714	54.9
G20501629	*E. hormaechei*	64	4794339	55.1
G20501634	*E. hormaechei*	63	4864880	55.4
G20501636	*E. hormaechei*	47	4922294	54.9
G20501642	*E. hormaechei*	51	4793983	55.4
G20501643	*E. hormaechei*	23	4557972	55.6
G20501644	*E. hormaechei*	27	4558600	55.6
G20501645	*E. hormaechei*	24	4558110	55.6
G20501646	*E. hormaechei*	24	4557266	55.6
G20501648	*E. hormaechei*	24	4557330	55.6
G20501649	*E. hormaechei*	24	4558124	55.6
G20501651	*E. hormaechei*	26	4557835	55.6
G18503208	*E. hormaechei*	40	4833404	55.6
G20500758	*E. hormaechei*	112	5244682	54.8
G20501620	*E. asburiae*	44	4792943	55.7
G20501630	*E. hormaechei*	51	4920922	54.9
G20501658	*E. bugandensis*	29	4766543	56.0
G20501659	*E. hormaechei*	87	4860737	55.3
G18503414	*E. cloacae*	37	5022229	54.8
G18653032	*E. cloacae*	119	5023340	54.4
G18653131	*E. cloacae*	128	5025193	54.4
G20500018	*E. cloacae*	70	4863949	54.9
G20500019	*E. cloacae*	36	4904377	54.9
G20500020	*E. cloacae*	35	4903831	54.9
G20500031	*E. cloacae*	70	4864760	54.9
G20500115	*E. cloacae*	38	4902900	54.9
G20500540	*E. cloacae*	51	5098049	54.8
G20500541	*E. cloacae*	59	5198147	54.6
G20500548	*E. cloacae*	78	5186423	54.7
G20500549	*E. cloacae*	72	5186354	54.7
G20500550	*E. cloacae*	72	5186743	54.7
G20500551	*E. cloacae*	70	5186208	54.7
G20500552	*E. cloacae*	73	5186627	54.7
G20500553	*E. cloacae*	72	5186184	54.7
G20500554	*E. cloacae*	73	5186653	54.7

G20500555	*E. cloacae*	72	5186183	54.7
G20500757	*E. cloacae*	66	5261469	54.2
G20500764	*E. cloacae*	78	5401537	54.4
G20500765	*E. cloacae*	122	5387618	54.4
G20500767	*E. cloacae*	64	4996754	54.9
G20500770	*E. cloacae*	105	4902478	54.9
G20501632	*E. cloacae*	45	5204392	54.7
G20501633	*E. cloacae*	43	5203940	54.7
G20501655	*E. cloacae*	110	5083725	54.8
G18653134	*E. roggenkampii*	22	4719711	56.0
G18653132	*E. roggenkampii*	24	4719347	56.0
G20500088	*E. roggenkampii*	17	4658365	56.0
G20500538	*E. roggenkampii*	111	5230556	55.3
G18600051	*E. bugandensis*	27	4530193	56.2
G18600052	*E. bugandensis*	26	4657657	56.1
G20501631	*E. kobei*	81	4865737	55.5
G20501635	*E. kobei*	66	4995558	54.8
G20500030	*E. cancerogenus*	15	4829897	55.5

Table S3: Initial identification of *Enterobacter hormaechei* by reference and sentinel laboratories.

ID	Initial sentinel lab ID	VITEK2 species	% probability
UCH-OGU-P1593	*Pseudomonas aeruginosa*	*Pseudomonas aeruginosa*	missing
UCH-OGU-P1573	*Pseudomonas aeruginosa*	*Pseudomonas aeruginosa*	98%
UCH-OGU-P01062	*Enterobacteriaceae*	*Enterobacter cloacae complex*	98%
UCH-OGU-P549A	*Enterobacteriaceae*	*Enterobacter cloacae complex*	missing
UCH-OGU-P0547A	*Enterobacteriaceae*	*Enterobacter cloacae complex*	96%
UCH-OGU-P0656	*Enterobacteriaceae*	*Enterobacter cloacae*	95%
UCH-OGU-P0267C	*Enterobacteriaceae*	*Enterobacter cloacae*	99%
UCH-OGU-P0144A	*Enterobacteriaceae*	*Enterobacter cloacae*	98%
UCH-OGU-P1581A	*Enterobacteriaceae*	*Enterobacter cloacae*	*missing*
UCH-OGU-P1521	*Klebsiella pneumoniae*	*Klebsiella pneumoniae*	*missing*
UCH-OGU-P1193	*Enterobacter cloacae*	*Enterobacter cloacae*	*99%*
UCH-OGU-P2006	*Enterobacter cloacae*	*Enterobacter cloacae*	*99%*
UCH-OGU-P1377	*Enterobacter cloacae*	*Enterobacter cloacae*	*99%*
UCH-OGU-P1394	*Enterobacter cloacae*	*Enterobacter cloacae*	*99%*
UCH-OGU-P1267	*Enterobacter cloacae*	*Enterobacter cloacae*	*missing*
UCH-OGU-P0144B	*Acinetobacter baumannii*	*Acinetobacter baumannii*	*missing*
UCH-OGU-P0144C	*Acinetobacter baumannii*	*Acinetobacter baumannii*	*94%*
OAU-OFO-542	*Klebsiella pneumoniae*	*Escherichia coli*	*96%*
OAU-AAA-578	*Enterobacteriaceae*	*Salmonella*	*93%*
OAU-OFO-129A	*Enterobacteriaceae*	*Escherichia coli*	*missing*
OAU-OFO-575i	*Enterobacteriaceae*	*Escherichia coli*	*95%*
UCH-OGU-P1581B	*Enterobacteriaceae*	*Escherichia coli*	*missing*
LUT-BC-316	*Staphylococcus aureus*	*Enterobacter aerogenes*	*missing*
OSO-OJO-S18	*Streptococcus pyogenes*	*Escherichia coli*	*missing*
UCH-OGU-P1394	*Klebsiella pneumoniae*	*Escherichia coli*	*98%*
UCH-OGU-P1730	*HVN*	*Escherichia coli*	*missing*

LUT-BC-267	*Acinetobacter baumannii*	*Staphylococcus haemolyticus*	*missing*
OSO-OJO-E2	*Pseudomonas aeruginosa*	*Escherichia coli*	*98%*
UCH-OGU-P1579i	*Escherichia coli*	*Escherichia coli*	*99%*
UCH-OGU-19-P1403	*Escherichia coli*	*Enterobacter cloacae*	99%
UCH-OGU-19-P1400B	*Staphylococcus aureus*	*Enterobacter cloacae*	99%
LUT-BC-19-196	*Pantoea agglomerans*	*Enterobacter cloacae*	97%
LUT-BC-19-506	*Coagulase-negative staphylococcus*	*Enterobacter cloacae*	99%
LUT-BC-19-584	*Escherichia coli*	*Enterobacter cloacae*	99%
OAU-OA-144	*Enterobacter cloacae*	*Escherichia coli*	99%
ELL-NOO-157B	*Klebsiella pneumoniae*	*Enterobacter cloacae*	93%
ELL-NOO-172A	*Klebsiella pneumoniae*	*Enterobacter cloacae*	94%
ELL-NOO-171B	*Klebsiella pneumoniae*	*Enterobacter cloacae*	94%
ILO-20-ET-013	*Escherichia coli*	*Enterobacter cloacae*	98%
OAU-OA-013	*Escherichia coli*	*Enterobacter cloacae*	99%
OAU-LT-116	*Escherichia coli*	*Enterobacter cloacae*	99%
OAU-OA-020	*Klebsiella pneumoniae*	*Enterobacter cloacae*	98%
CLL-NOO-303	*Klebsiella pneumoniae*	*Enterobacter cloacae*	93%
ELL-NOO-81Bi	*Klebsiella pneumoniae*	*Enterobacter cloacae*	98%
ELL-NOO-83B	*Klebsiella pneumoniae*	*Enterobacter cloacae*	98%
ELL-NOO-83Bd	*Klebsiella pneumoniae*	*Enterobacter cloacae*	99%
ELL-NOO-92A	*Klebsiella pneumoniae*	*Enterobacter cloacae*	99%
ELL-NOO-97B	*Klebsiella pneumoniae*	*Enterobacter cloacae*	98%
ELL-NOO-114B	*Klebsiella pneumoniae*	*Enterobacter cloacae*	96%

Table S4: Vitek2 test specificity, sensitivity, positive predictive values, and negative predictive values

	E. hormaechei	*E. cloacae*
% sensitivity	0.0	1
% specificity	0.0	0.0
% positive predictive value	0.0	1
% negative predictive value	0.0	0.0

Table S5: Numbers of Enterobacter genomes and AmpC variants (using AMRFinderPlus and CARD)

Enterobacter spp.	Number Of genomes	Number of AmpC variants	Assigned ACT, CMH, and MIR variant(s)
E. hormaechei	61	14	ACT-15(4), -16(8), -17(2), -24(4), -25(11), -41(1), -45(6), -46(4), -61(2)-69(2) -70(1), -74(7) -75(5), -84(4)
E. cloacae	26	3	CMH-1(1), -3(7), -4(15), -7(3)
E. roggenkampii	4	3	MIR-2(2), -23(1), ACT-62(1),
E. bugandensis	3	3	ACT-72(1), -77(1), -78(1)
E. kobei	2	2	ACT-28(1), ACT-9(1)
E. asburiae	1	1	ACT-2(1)
E. cancerogenous	1	1	ACT-8(1)

*The number of strains with each variant is in brackets

Table S6: Antimicrobial Resistance Phenotypic and Genotypic Concordance Analysis

	E. hormaechei			*E. cloacae*		
Drug	concordance	specificity	sensitivity	concordance	specificity	sensitivity
Ampicillin	1	NA	1	1	NA	1
Amoxicillin/Clavulanic Acid	0.652173913	NA	0.652174	0.933333	0	1
Piperacillin/Tazobactam	0.538461538	0.416667	0.642857	0.4	0	1
Cefuroxime	1	NA	1	0.733333	NA	0.733333
Cefuroxime Axetil	1	NA	1	0.733333	NA	0.733333
Ceftriaxone	0.72	0	1	0.866667	0.75	0.909091
Cefoperazone/Sulbactam	0.307692308	0	1	0.266667	0.266667	NA
Cefepime	0.423076923	0	1	0.733333	0.5	0.888889
Ertapenem	0.869565217	1	0.4	-	-	-
Imipenem	1	1	1	-	-	-
Meropenem	1	1	1	-	-	-
Amikacin	1	1	1	0.333333	0.333333	NA
Gentamicin	0.923076923	0.888889	0.941176	1	1	1
Colistin	0.75	1	0	0	NA	0
Trimethoprim/Sulfamethoxazole	0.833333333	0.6	1	1	1	1

Table S7: Antimicrobial Susceptibility Testing (AST) data of outbreak strains

ST109-NDM	Alternative ID	Amoxicillin/Clavulanic Acid	Piperacillin/Tazobactam	Cefuroxime	Cefuroxime Axetil	Ceftriaxone	Cefoperazone/Sulbactam	Cefepime	Ertapenem	Imipenem	Meropenem	Amikacin	Gentamicin	Nalidixic Acid	Ciprofloxacin	Tigecycline	Nitrofurantoin	Trimethoprim/Sulfamethoxaz
UCH-OGU-P0144A	G18503214	>= 32/R	>= 128/R	>= 64/R	>= 64/R	>= 64/R	>= 64/R	16/R	>= 8/R	>= 16/R	>= 16/R	>= 64/R	>= 16/R	>= 32/R	<= 0.25/R	<= 0.5/R	64/I	<= 20/S
UCH-OGU-P0656	G18503210	>= 32/R	>= 128/R	>= 64/R	>= 64/R	>= 64/R	>= 64/R	8/R	>= 8/R	>= 16/R	>= 16/R	>= 64/R	>= 16/R	>= 32/R	<= 0.25/S	1/S	64/I	<= 20/S
UCH-OGU-P0144C	G18581058		>= 128/R			>= 64/R	>= 64/R	16/I		>= 16/R	>= 16/R		>= 16/R		<= 0.25/R	<= 0.5/S		<= 20/S
ST148																		
UCH-OGU-P01062	G18503206	>= 32/R	16/S	>= 64/R	>= 64/R	>= 64/R	<= 8/S	4/S	<= 0.5/S	1/S	<= 0.25/S	<= 2/S	>= 16/R	16/S	1/S	2/S	64/I	>= 320/R
UCH-OGU-P1581B	G18593207	>= 32/R	32/I	>= 64/R	>= 64/R	>= 64/R	<= 8/S	2/S	<= 0.5/S	1/S	<= 0.25/S	<= 2/S	>= 16/R	16/S	1/S	2/S	128/R	>= 320/R
UCH-OGU-P0267C	G18503213	>= 32/R	32/I	>= 64/R	>= 64/R	>= 64/R	<= 8/S	2/S	<= 0.5/S	0.5/S	<= 0.25/S	<= 2/S	>= 16/R	16/S	1/S	2/S	64/I	>= 320/R
UCH-OGU-P1573	G18501064		32/S				<= 8/S	32/R		0.5/S	<= 0.25/S	4/S	>= 16/R		1/S	1/R		
UCH-OGU-P1593	G18501062		<= 4/S				<= 8/S	<= 1/S		1/S	<= 0.25/S	<= 2/S	<= 1/S		<= 0.25/S	1/R		
